# A rebinding-assay for measuring extreme kinetics using label-free biosensors

**DOI:** 10.1038/s41598-021-87880-x

**Published:** 2021-04-15

**Authors:** John G. Quinn

**Affiliations:** Biophysical Group, Biochemical and Cellular Pharmacology, Genentech Inc., 1 DNA Way, South San Francisco, CA 94080 USA

**Keywords:** Biophysical chemistry, Computational biophysics, Kinetics, Mathematics and computing, Computational science, Molecular medicine, Fluid dynamics, Techniques and instrumentation

## Abstract

In vitro kinetic measurements allow mechanistic characterization of binding interactions and are particularly valuable throughout drug discovery, from confirmation of on-target binding in early discovery to fine-tuning of drug-binding properties in pre-clinical development. Early chemical matter often exhibits transient kinetics, which remain challenging to measure in a routine drug discovery setting. For example, characterization of irreversible inhibitors has classically relied on the alkylation rate constant, yet this metric fails to resolve its fundamental constituent rate constants, which drive reversible binding kinetics and affinity complex inactivation. In other cases, extremely rapid association processes, which can approach the diffusion limit, also remain challenging to measure. To address these limitations, a practical kinetic rebinding assay is introduced that may be applied for kinetic screening and characterization of compounds. The new capabilities afforded by this probe-based assay emerge from mixed-phase partitioning in a flow-injection configuration and have been implemented using label-free biosensing. A finite element analysis-based biosensor model, simulating inhibition of rebinding within a crowded hydrogel milieu, provided surrogate test data that enabled development and validation of an algebraic model for estimation of kinetic interaction constants. An experimental proof-of-principle demonstrating estimation of the association rate constant, decoupled from the dissociation process, provided further validation.

## Introduction

In vitro kinetic assays are valuable throughout drug discovery, particularly during hits-to-leads progression, by providing mechanistic discrimination of artifactual binding^1–3^ from tractable binding modes. Routine kinetic measurements also allow compounds to be optimized towards a desirable target-specific kinetic profile^4–6^ allowing fine tuning of compound properties, including target engagement and residence time for enhanced clinical efficacy^7^. Indeed, routine measurement of direct binding kinetics using real-time label-free biosensors^8, 9^ provides a practical means of leveraging kinetics for compound prioritization yet the transient kinetics of early chemical matter largely remains beyond the limit of detection. To date, transient kinetics, defined here as affinity complexes that fully dissociate in < 1 s, are measured using low throughput stop-flow based methods^10^, which are impractical for analysis of compound collections in a drug discovery setting. A biosensor-based approach has recently been reported^11^, addressing this limitation but specific system customizations are required to enable routine application. Probe-based kinetic competition assays^12^ may be implemented using surface plasmon resonance (SPR)-based biosensors^13^, or other equivalent flow-injection-based biosensors such as grating coupled interfereometry^14^. However, the estimation of transient kinetics and extremely rapid association processes (i.e. association rate constant (*k*_*a*_) exceeds 5 × 10^7^ M^−1^ s^−1^) for small molecule inhibitor interactions remains challenging using such biosensors^15^ and here we introduce a probe-based kinetic rebinding assay to address this unmet need. The rebinding assay is relatively insensitive to both bulk-refractive index mismatches and baseline drift and may be particularly valuable for estimating extreme kinetics of mechanistically complex inhibitors such as irreversible inhibitors. Biophysically realistic virtual biosensors^11, 15, 16^ built using finite element analysis-based computational modeling have provided realistic surrogate data for validation of mechanistic binding models in the past and was adopted for development and validation of the rebinding assay. Advection in bulk flow and diffusion/reaction within the extended hydrogel matrix were modeled as coupled domains of defined volume, where species advection and reaction were computed. Importantly, a finite element-based biosensor model is far too mathematically complex to allow practical estimation of kinetics from actual experimental data. Instead, the virtual instrument simulated rebinding progress curves over extensive parameter ranges to produce surrogate experimental data that enabled the development and validation of an algebraic model. Non-linear least-squares fitting of the algebraic model to both surrogate experimental data, and actual experimental data, returns reliable estimates of the kinetic interaction constants without requiring knowledge of biophysical parameters beyond those routinely available in a practical drug discovery setting (e.g. analyte concentration, surface binding capacity and molecular weight).

## Results

### Principle of rebinding-assay

**Figure 1:**
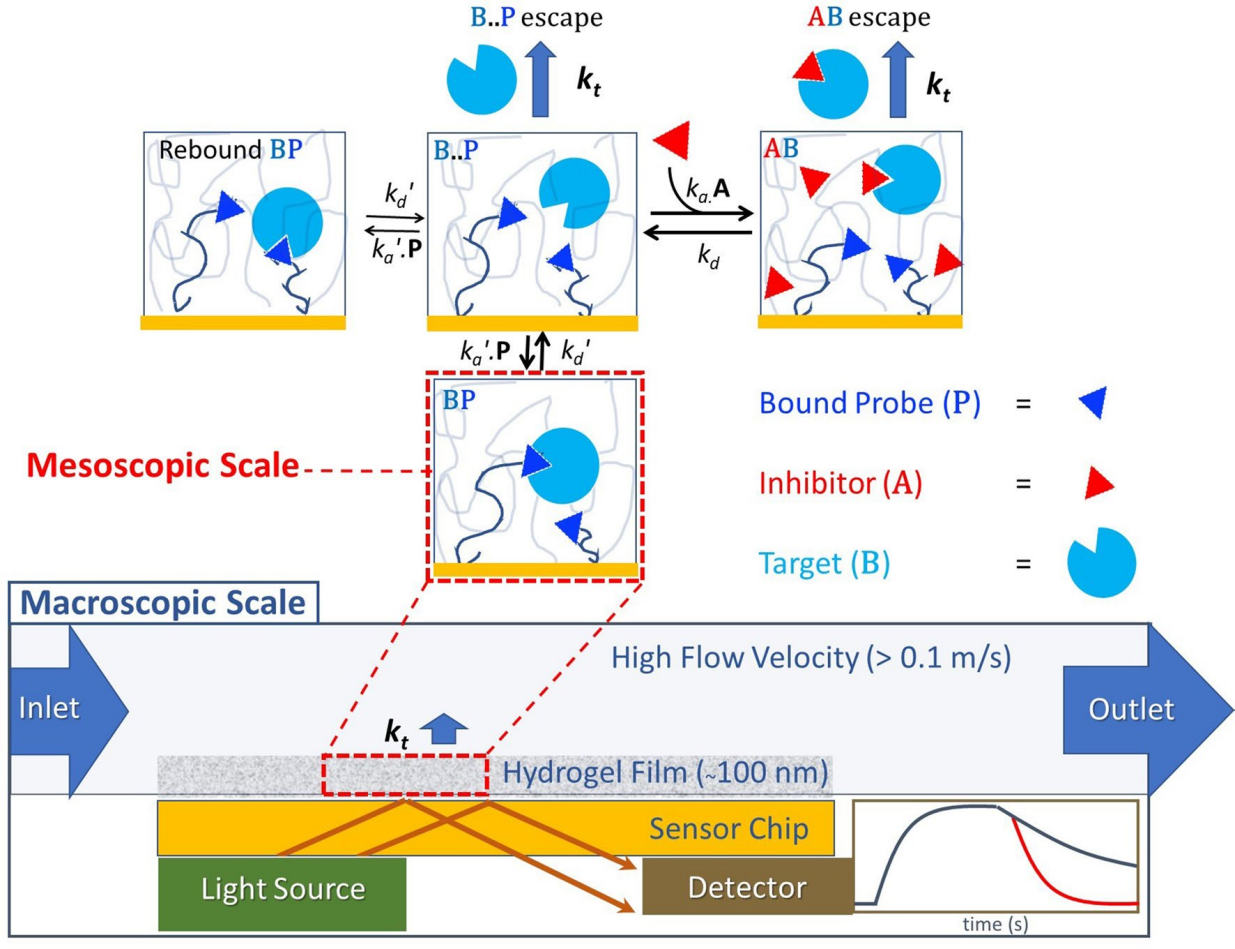
Reaction/diffusion pathways of the rebinding-assay for a flow injection-based biosensor. A macroscopic scale view (bottom) depicts a hydrogel bound to a sensor chip and interrogated by a label-free detector to produce a binding progress curve with (red), and without (blue), inhibition of rebinding during the dissociation phase. A series of mesoscopic scale views of the hydrogel (four upper panels) depict the reaction pathways. The hydrogel polymer is depicted here as random loops derivatized to possess a high concentration of **P** (blue triangle), which is competitive with inhibitor **A** (red triangle) in binding to target **B** (blue circle). Paired forward- and reverse-rate constants for formation of **BP** and **AB** are *k*_*a*_*′* (M^−1^ s^−1^)/* k*_*d*_*′* (s^−1^) and *k*_*a*_ (M^−1^ s^−1^)/ *k*_*d*_ (s^−1^), respectively, while *k*_*t*_ (s^−1^) is the mass transport coefficient that defines the hydrogel escape rate. The molecular contacts stabilizing **BP** dissociate releasing **B** to become a transient unbound species **B..P** that can readily reform **BP**, pairing with any **P** in its vicinity. Multiple rebinding cycles are favored at higher concentrations of **B** and high *k*_*a*_*′* before **B** escapes the sensing region at rate *k*_*t*_. Injected **A** sequesters **B** depleting **B..P** to form **AB**, which in turn exits the hydrogel thereby negating rebinding. However, in the case that **AB** dissociates before exiting, then liberated **B** will revert to **B..P**. Although not depicted here, **B** that has escaped the hydrogel has a probability of re-entering the hydrogel further downstream replenishing **B..P**.

The flow injection-based biosensor system and associated reaction/diffusion pathways for the rebinding assay are illustrated in Fig. 1 and were modeled using a finite element analysis-based computational model in order to produce surrogate experimental data to develop, and validate, an algebraic model suitable for estimation of kinetic constants from inhibition of rebinding curves. Three species of biomolecule are required, namely a probe, inhibitor and target, and may be chosen from almost any class of biomolecule, assuming that competition exists between the inhibitor and probe in binding target. Surrogate experimental data was produced from a virtual instrument by solving sets of coupled partial differential equations using a finite element analysis engine (see Method section for details). The flux balance in **B** for surface reaction relative to mass transport from bulk liquid determines the degree of mass transport limitation, which slows binding and promotes rebinding, and may be expressed by the dimensionless Damköhler number (Da), expressed here in terms of biosensor response as

1 $${\text{Da }} = k_{a}^{\prime } \cdot \left( {{\text{R}}_{{{\text{max}}}} - {\text{R}}} \right){/}k_{t}$$

where R_max_ − R ∝ **P**, R_max_ is the biosensor response at surface saturation, R is the response at any given time and *k*_*t*_ (s^−1^) is the mass transport coefficient^17^. *k*_*t*_ defines the rate at which **B** may enter, or escape, the mass transport boundary layer that forms over the sensing region. The escape time τ = 1/*k*_*t*_ is typically in the μs-to-ms regime and confers extraordinary sensitivity to transient kinetics in an inhibition of rebinding format. An expression for *k*_*t*_ that accounts for mass transport resistance through the flow cell and through the hydrogel may be defined by the expression

2 $$k_{t} = {\text{T}}_{{\upgamma }} { } . 1.281 .\left( {\frac{{\nu_{c} {\text{.D}}}}{{2.{\text{h}}.{\text{l}}}}} \right)^{1/3}$$

where ν_c_ = maximum flow velocity at center of flow channel (m/s), D = diffusion coefficient of analyte in bulk liquid (m^2^/s), h = flow cell height (m) and l = length (m) of functionalized sensing region upstream and including the optically interrogated region. The incorporation of a hydrogel transport resistance term T_γ_ is required to account for hydrogel transport resistance^18^. This term is defined by the height of the hydrogel H_gel_ relative to the mean free path taken by **B** before being bound, where T_γ_ = Tanh(γ)/γ with γ = H_gel_ / (D_gel_.K_part_/(*k*_*a*_*′*.**P**))^0.5^, K_part_ = hydrogel partition coefficient (unit less) and D_gel_ = diffusion coefficient of **B** within the hydrogel (m^2^/s). The mass transport coefficient may be expressed in terms of biosensor response as *k*_*t*_′ = 10^9^.M_r_B_.*k*_*t*_, where 10^9^ is a unit scaling factor (g m/mol) and M_r_B_ is the molecular weight of **B** (g/mol).

**Figure 2:**
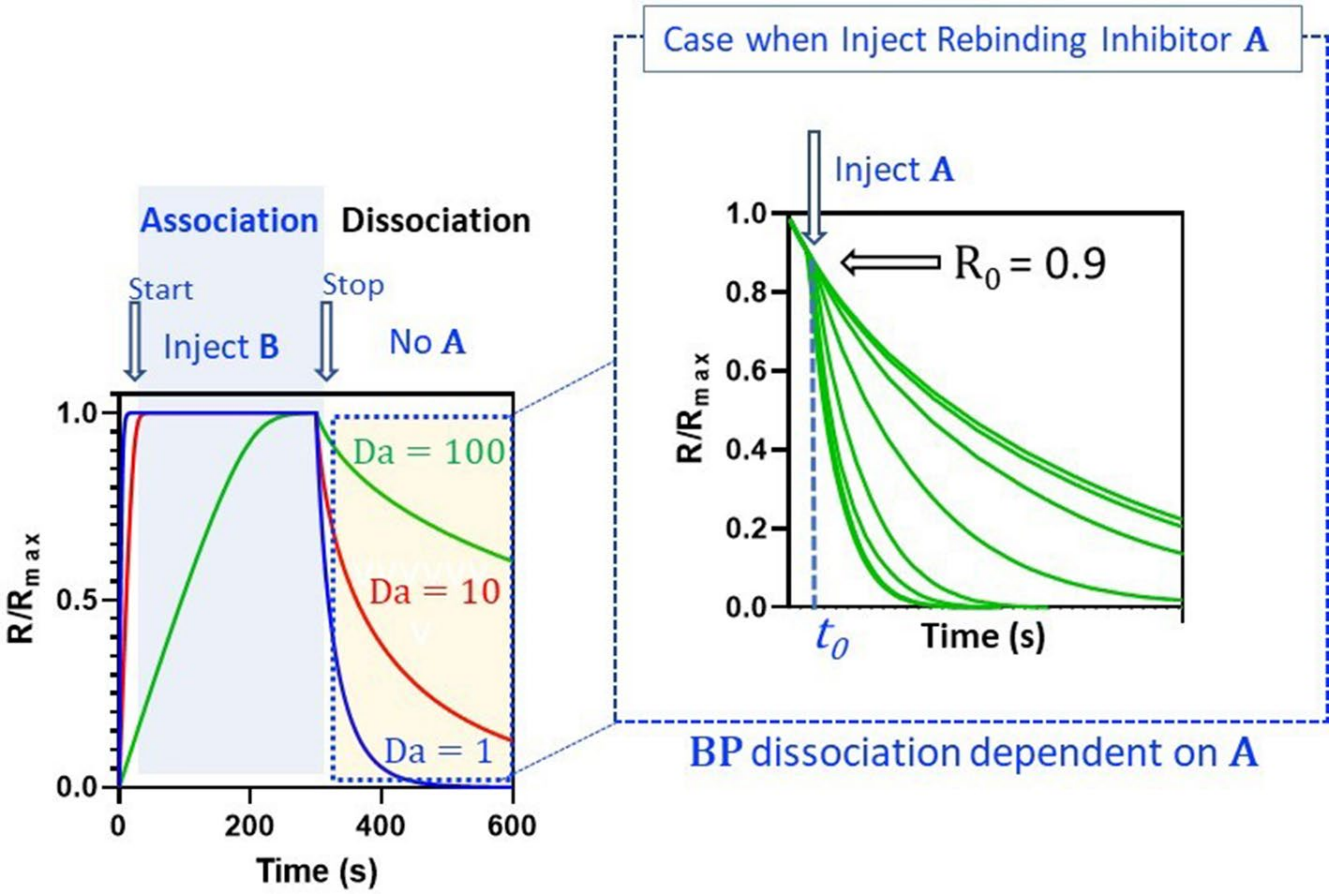
Simulated binding response curves for rebinding and inhibition of rebinding. Three mass transport limited binding curves for 10 nM **B** binding to **P** tethered within a hydrogel are shown on the left. The response curves were normalized to the maximum saturation response R_max_. The three simulated curves correspond to *k*_*t*_ values of 1 × 10^7^ s^−1^, 1 × 10^8^ s^−1^ and 1 × 10^9^ s^−1^ producing Da values of 100, 10, and 1, respectively. The simulation was performed using a two-compartment model (see Methods for more details) and the interaction parameters were R_max_ = 20 RU, *k*_*a*_*′* = 5 × 10^7^ M^−1^ s^−1^ and *k*_*d*_*′* = 0.05 s^−1^. Inhibition of rebinding during the dissociation phase at fixed operating conditions is depicted on the right, where **A** was injected (injection time = t_0_) at 100 µM and replicated over a serial fivefold range in *k*_*a*_ from 1 × 10^5^ M^−1^ s^−1^ to 7.81 × 10^9^ M^−1^ s^−1^ to produce dose-dependent inhibition of rebinding. The response observed at the onset (t_0_) of the inhibitor injection is R_0_. Inhibition curves were simulated using the virtual instrument and finite element analysis engine described in the Method section.

Equations (1) and (2) can be used to guide experimental design and imply that rebinding increases with increasing *k*_*a*_*′*, increasing **P**, increasing hydrogel thickness and decreasing flow rate. Equation (2) allows approximation of *k*_*t*_, where hydrogel parameters (e.g. H_gel_, D_gel_, K_part_) are available, although it is generally estimated as a globally constrained parameter when fitting a mechanistic kinetic model to binding progress curves^15^. The simulated binding curves in Fig. 2 (plot to the left) show that mass transport resistance results in slowing of both association- and dissociation-phase curves as a function of increasing mass transport resistance i.e. increasing Da. However, injection of an excess of **A** during the dissociation phase (Fig. 2 (plot on right)) inhibits rebinding of **B**, restoring the true dissociation rate of **AB**, while partial inhibition occurs at lower concentrations of **A**.

### Inhibition of rebinding model

**Figure 3:**
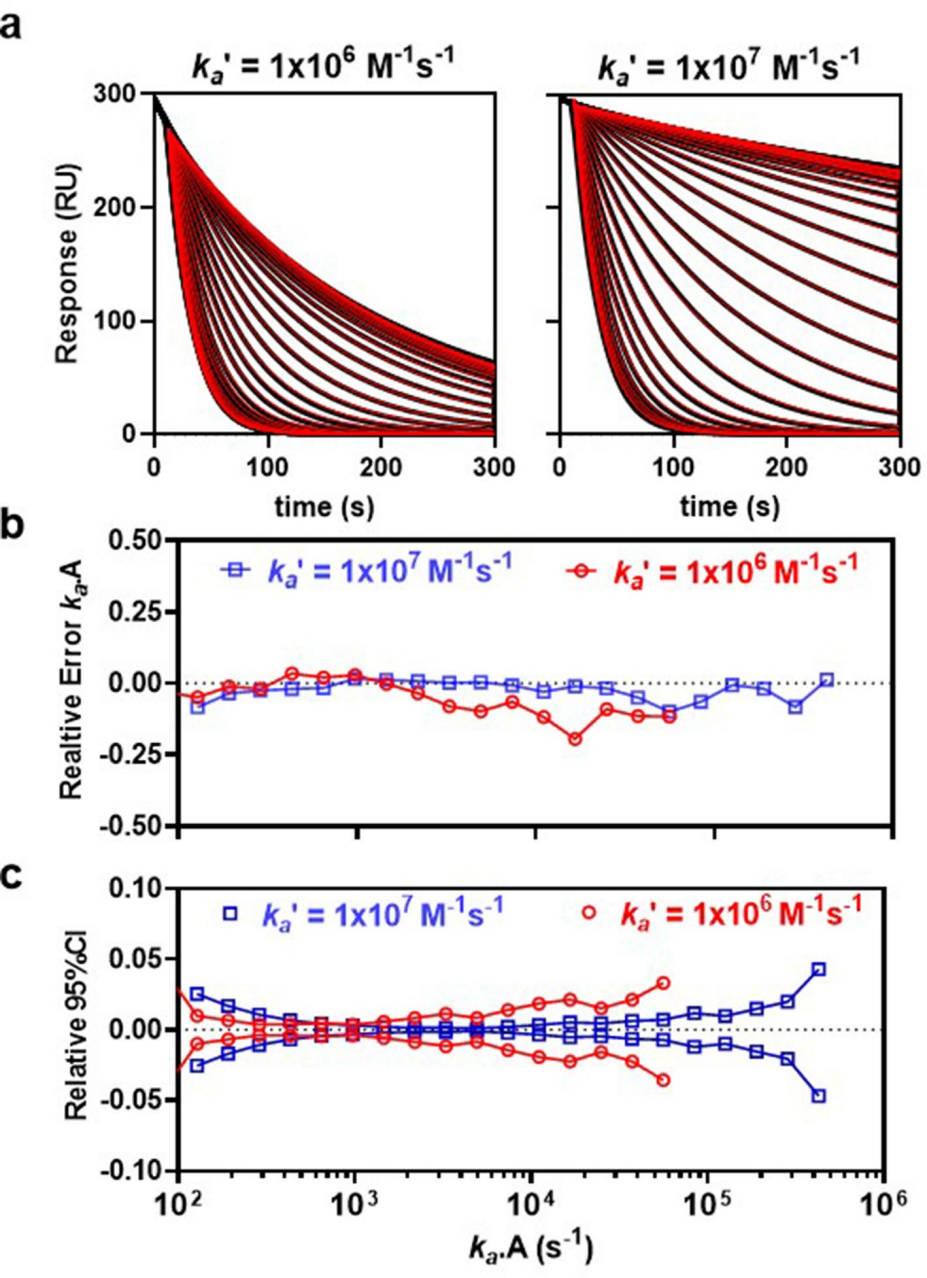
Estimation of *k*_*a*_ from individual inhibition curves at two values of *k*_*a*_*′*. (**a**) Fitted inhibition curves for moderate- (left panel Da = 8.7) and high- (right panel, Da = 87) mass transport limitation. Inhibition curves (black) were fit (red) to Eq. (4), where *k*_*a*_ was fit locally, giving independent estimates of *k*_*a*_ for each curve, while all other parameters were held constant. (**b**) Relative error in *k*_*a*_*.A* as a function of true *k*_*a*_*.A*. All values where the absolute relative error was < 0.2 are shown. Relative error = 1-(true_*k*_*a*_.**A**/*k*_*a*_.**A**). (**c**) 95% confidence intervals expressed as a fraction of estimated *k*_*a*_*.A* and plotted versus true *k*_*a*_*.A* and were < 0.05. Details of the numerical simulations in (**a**) are as follows. At t = 0 s, **B** is pre-bound to the hydrogel to a concentration equivalent to R_0_ = 300 RU = 0.1R_max_. At t = 10 s, 0.5 mM **A** is injected inhibiting rebinding of **B** thereby accelerating escape of **B**. The inhibition curves were simulated over serial 1.5-fold increasing *k*_*a*_ and included a blank inhibition curve, where **A** = 0. Other simulation parameters were as follows, *k*_*d*_ = 0.001 s^−1^, *k*_*d*_*′* = 0.05 s^−1^, h = 10 μm and ν_c_ = 0.15 m/s. Inhibition curves were simulated using the virtual instrument and finite element analysis engine described in the Method section.

An optimal balance between kinetic measuring range and sensitivity to inhibition requires relatively low levels of transport limitation (Da < 10) which also maintains monophasic-exponential behavior, as shown in the simulated inhibition of rebinding cures in Fig. 3a. Such monotonic behavior also depends on rapid development of a quasi-steady-state with respect to competing pathways acting upon **B..P**. Free **B** is assumed to be in an unbound transient state **B..P** within the hydrogel that is partitioned by three rate coefficients re-association *k*_*a*_*′.P* (s^−1^), inhibition *k*_*a*_*.A* (s^−1^) and hydrogel escape *k*_*t*_ (s^−1^). Therefore, under quasi-steady-state conditions the inhibition curves follow an approximate exponential decay where the change in response is given as

3 $$R = R_{0} .e^{{ - k_{off} .. t }}$$

where *k*_*off*_ (s^−1^) is the observed dissociation rate constant.

Injection of **A** produces an inhibition rate that increases *k*_*off*_ by lowering rebinding such that *k*_*off*_ ≈ *k*_*d*_*′* when fully inhibited. Rather than attempting to estimate transport and reaction fluxes from first principles, the transition state-based model described in Fig. 1 employs a phenomenological rebinding factor α, where *k*_*off*_ = * k*_*d*_*′.*α and is suitable for estimation of kinetic constants by fitting

4 $${\text{R }} = {\text{ R}}_{0} .{\text{e}}^{{ - k_{d}^{^{\prime}} .\alpha .{\text{t }}}}$$

where $$\alpha = \frac{\beta }{{ \beta + k_{a}^{\prime } \cdot {\varvec{P}}}}$$ and *β* = *k*_*t*_ + f.*k*_*a*_.**A**, f = 1/(1 + *k*_*d *_/*k*_*t*_), **P** = R_max_/G.M_r_B_.

The rebinding factor α is the degree to which dissociation is slowed due to rebinding and is given by the ratio of rebinding *k*_*a*_*′.P* relative to hydrogel escape β. Therefore, α = 1 when rebinding does not exist, or when rebinding is fully inhibited, otherwise α < 1. The partition function f accounts for loss in inhibition of rebinding due to unbinding of **AB** before escaping the hydrogel. The rate constants associated with f are high relative to *k*_*d*_*′* allowing a quasi-steady-state to be assumed. The response-to-concentration factor G expresses R_max_ in terms of a concentration of **P** and for many SPR-based biosensors G = 100 RU/g/L. The protein is assumed to be distributed homogenously within the hydrogel and there are experimental methods^19^ to estimate this parameter for higher accuracy. In practice, **P** is selected to possess moderate *k*_*d*_*′*, where 1 s ≤ 1/* k*_*d*_*′* ≤ 300 s, in order to support higher throughput. The assay tolerates wide variation in *k*_*a*_*′* since the reaction flux (*k*_*a*_*′.P*) may be modulated by the concentration of **P** yet higher values (e.g. *k*_*a*_*′* > 1 × 10^5^ M^−1^ s^−1^) are advantageous as this reduces the concentration of **B** required to achieve a given occupancy.

### Estimation of *k*_*a*_ for non-transient inhibitors

When analyzing non-transient binders we assume *k*_*d*_ <  < *k*_*t*_ and therefore f ≈ 1 and can be neglected. The kinetics of **BP** formation, namely *k*_*a*_*′* and *k*_*d*_*′,* are predetermined by conventional direct binding kinetics at low surface density of probe prior to characterizing inhibitors. These kinetic constants are then held constant when analyzing dose-dependent inhibition curves, allowing *k*_*a*_, *k*_*d*_ and *k*_*t*_ to be readily determined by global fitting Eq. (4). Although not essential, a zero-inhibition curve, where **A** = 0, may be included to allow *k*_*t*_ to be estimated in the absence of inhibition, where α = *k*_*t*_ /(*k*_*t*_ + * k*_*a*_*′*.**P**). Pre-estimation of *k*_*a*_*′* and *k*_*d*_*′* by conventional direct binding kinetics may be avoided by employing the soluble probe as an inhibitor species in the rebinding assay while also maintaining it as the surface-bound probe. In this case, **AB** becomes a fully soluble form of **BP** and hence both *k*_*a*_ and *k*_*a*_*′* govern the same interaction occurring in homogenous phase and heterogeneous phase, respectively. The associated kinetic rate constants are related through molecular weight-dependent diffusion scaling, where *k*_*a*_*′* ≈ *k*_*a*_/( M_r_P_ / M_r_B_)^1/3^ and substitution into Eq. (4) allows *k*_*a*_*′*, *k*_*d*_*′* and *k*_*t*_ to be estimated from global fitting. These parameters are then held constant when fitting unmodified Eq. (4) to inhibition curves recorded for the inhibitor panel allowing estimation of *k*_*a*_. We generated surrogate experimental data over a wide range in *k*_*a*_, at a fixed concentration of **A**, in order to determine the relative error and confidence intervals associated with *k*_*a*_-estimation for non-transient inhibitors, where *k*_*d*_ <  < *k*_*t*_, producing the data show in in Fig. 3. For each curve set the upper and lower limit curves correspond to *k*_*off*_ at zero inhibition and *k*_*d*_*'* at full inhibition, respectively. These limits define a twofold wider response widow for a tenfold higher *k*_*a*_*′* leading to an increase in measuring range with increasing mass transport limitation (Fig. 3b,c).

### Experimental proof-of-principle for estimating *k*_*a*_ of non-transient inhibitors

**Figure 4:**
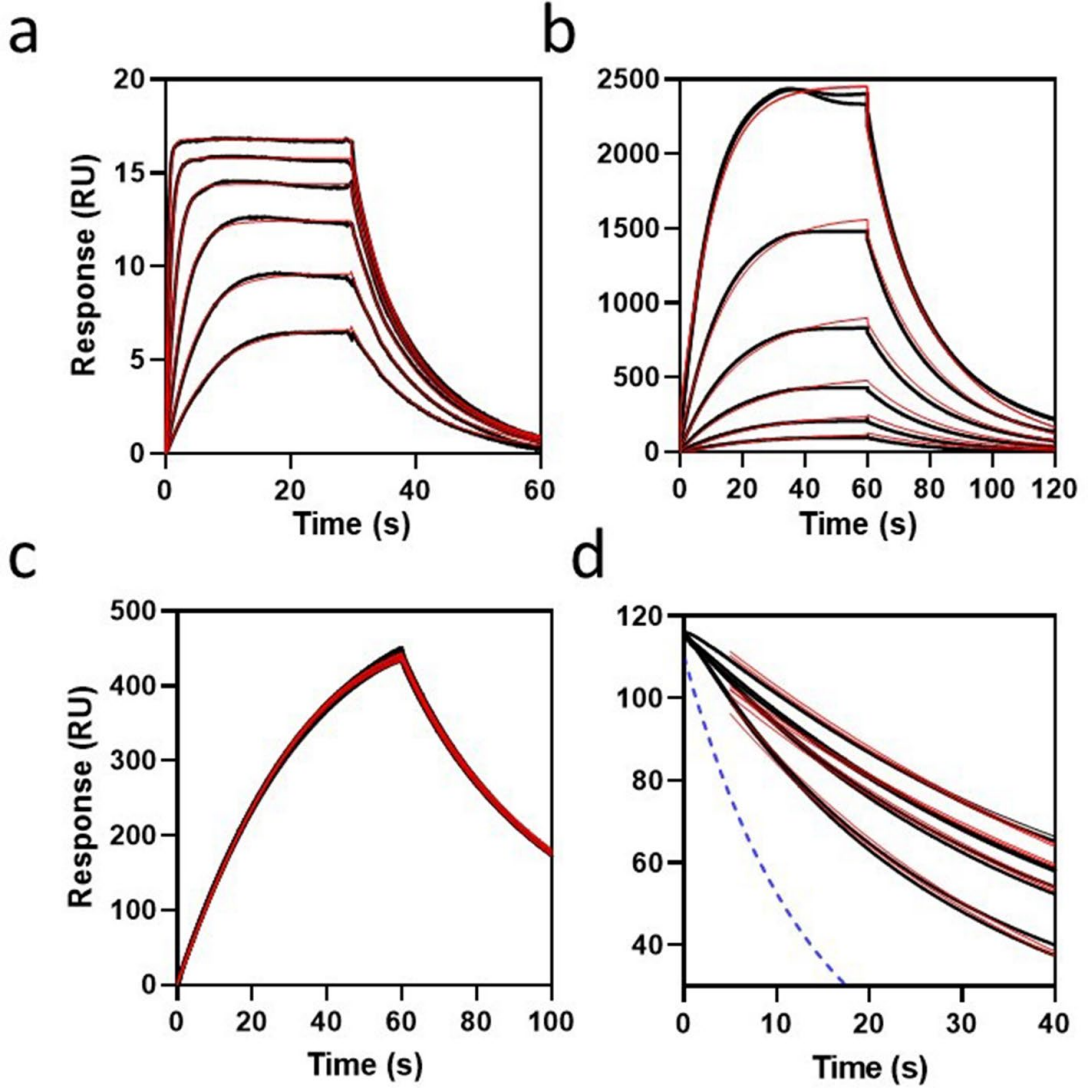
Experimental estimation of *k*_*a*_ using the rebinding assay. (**a**) Conventional SPR curves (black) for interaction of soluble probe with a hydrogel-bound target (M_r_ = 22 kDa) and fit to a two-compartment 1:1 interaction model (red) for estimation of *k*_*a*_ and *k*_*d*_. Probe (M_r_ = 737 Da) was injected for 30 s in duplicate over a serial doubling dilution range from 1 µM to 31 nM. All parameters were fit globally returning values of *k*_*a*_ = 2.76 ± 0.008 (× 10^6^) M^−1^ s^−1^, *k*_*d*_ = 0.125 ± 0.0003 s^−1^, R_max_ = 16.8 ± 0.01 RU and %χ^2^ = 0.13. (**b**) Conventional SPR curves (black) for interaction of soluble target with a hydrogel-bound probe and fit to a two-compartment 1:1 interaction model (red) for estimation of *k*_*a*_*′* and *k*_*d*_*′*. Target was injected for 60 s in duplicate over a serial-doubling dilution range from 1 µM to 31 nM. All parameters were fit globally returning values of *k*_*a*_*′* = 8.5 ± 0.004 (× 10^4^) M^−1^ s^−1^, *k*_*d*_*′* = 0.075 s^−1^, R_max_ = 4052 ± 0.001 RU and %χ^2^ = 0.31. (**c**) Estimation of R_max_ from the target-loading phase of rebinding curves. Briefly, 500 nM target was injected over a probe-coated surface (n = 8) at 30 μL/min for 60 s followed by dissociation. A simple 1:1 model was fit returning an estimate of the saturation response R_max_ = 1025 ± 10 RU. (**d**) Inhibition phase region of curves in (**c**) when dissociation was ≤ 118 RU which was the injection point for the rebinding inhibitor. The eight curves correspond to the late dissociation phase of the target-loading curves in (**c**) for duplicate injections of inhibitor at 0 μM, 0.016 μM, 0.6 μM and 4 μM. Curves (black) were time-normalized at 118RU and fit (red) to Eq. (4) returning *k*_*a*_ = 3.07 ± 0.01 (× 10^6^) M^−1^ s^−1^, *k*_*t*_ = 10.55 s^−1^ and %χ^2^ = 1.1, where *k*_*a*_*′*, *k*_*d*_*′*, *k*_*d*_ and R_max_ were held constant at the values estimated from (**a**), (**b**) and (**c**). R_0_ was fit locally and both *k*_*a*_ and *k*_*t*_ were fit globally. Note: Limited compound solubility prevented use of higher inhibitor concentrations.

The probe, in soluble form, was employed as a surrogate inhibitor in order to cross-validate parameter return. In this particular case, the homogenous phase *k*_*a*_ may be related to the heterogeneous phase *k*_*a*_*′* by normalizing for differences in collision frequency through diffusion rescaling, where *k*_*a*_*′* = *k*_*a*_/(M_r_P_/M_r_B_)^1/3^, and the experimental data is show in Fig. 4. Direct binding kinetics returned *k*_*a*_ = 2.76 ± 0.008 (× 10^6^) M^−1^ s^−1^ for soluble-probe binding to immobilized-target (Fig. 4a) and after diffusion re-scaling is *k*_*a*_ = 1.0 × 10^5^ M^−1^ s^−1^, which is within 15% of *k*_*a*_*′* = 8.5 ± 0.004 (× 10^4^) M^−1^ s^−1^ obtained for the reverse format (Fig. 4b), where soluble-target was bound to immobilized-probe. More importantly, *k*_*a*_ = 2.76 ± 0.008 (× 10^6^) M^−1^ s^−1^ for direct binding of probe and is within 10% of *k*_*a*_ = 3.07 ± 0.01 (× 10^6^) M^−1^ s^−1^ returned from inhibition of rebinding (Fig. 4d), thereby cross-validating the results from these reversed assay formats. The assay is compatible with a throughput of > 500 inhibitors/day using a biacore 8 K + (Cytiva Inc), assuming four concentrations per inhibitor and may be performed in singleton for higher throughput screening (e.g. fragment libraries).

### Estimation of transient kinetics

**Figure 5:**
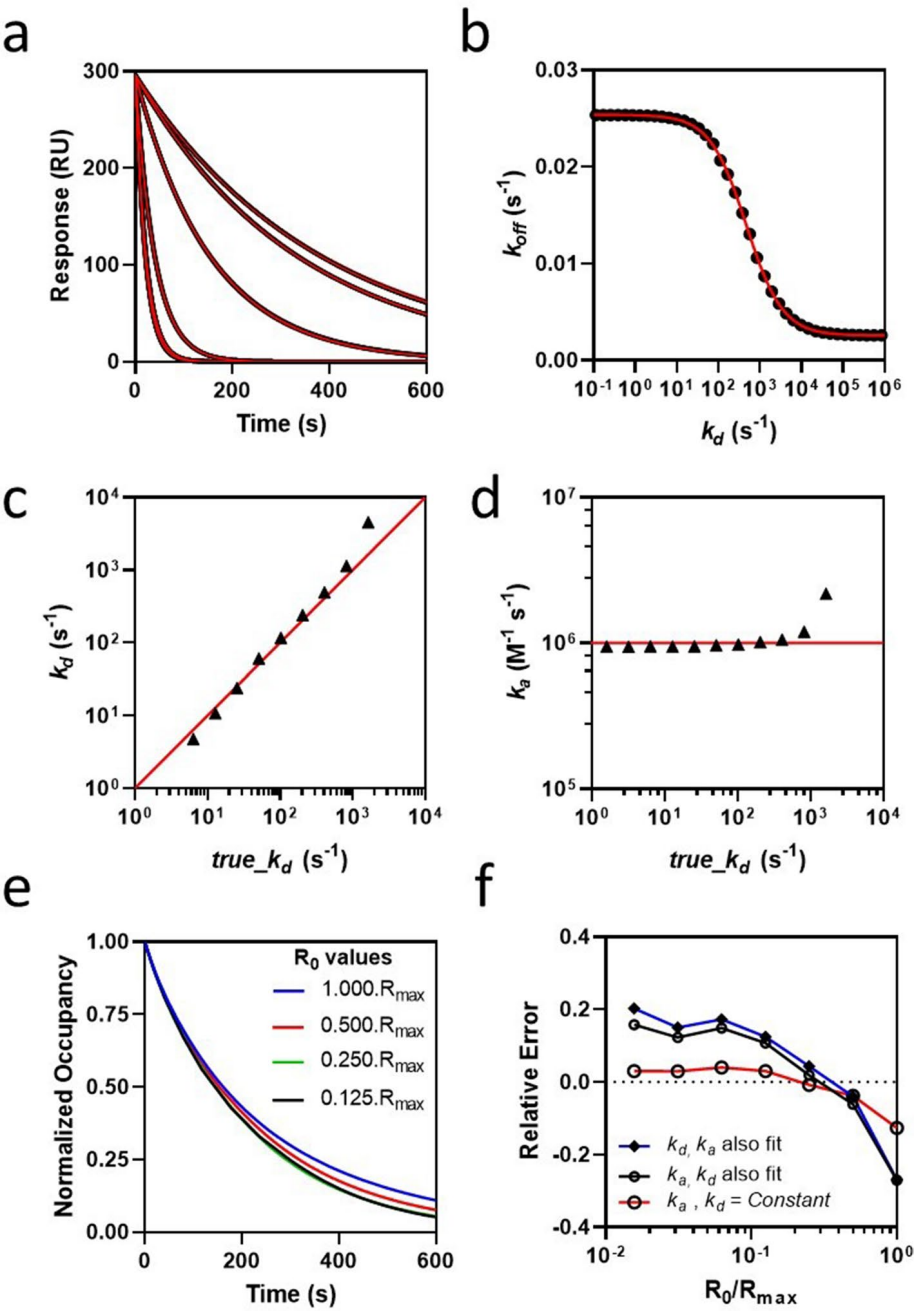
Kinetic detection limits for transient inhibitor binding. (**a**) Inhibition curves corresponding to *k*_*a*_ values of 10^4^, 10^5^, 10^6^, 10^7^, 10^8^ and 10^9^ (M^−1^ s^−1^) each with eight superimposable replicates corresponding to *k*_*d*_ (0.0, 0.1, 1.0, 10.0 (s^−1^)) all with, and without, inclusion of an irreversible adduct formation rate *k*_*inact*_ = 0 s^−1^, or 1 s^−1^, respectively. The degree of inhibition increased with increasing *k*_*a*_ and inhibition curves superimpose at full inhibition, corresponding to the two highest *k*_*a*_ values of 10^8^ M^−1^ s^−1^ and 10^9^ M^−1^ s^−1^, respectively. (**b**) Partition curve for loss of inhibition of rebinding as a function of transient *k*_*d*_, where the apparent dissociation rate constant *k*_*off*_ was obtained by fitting Eq. (3) to surrogate experimental data over a range in *k*_*d*_ , covered over a serial 1.5-fold range from 10^−1^ to 10^5^ for a single value of *k*_*a*_ = 1 × 10^6^ (M^−1^ s^−1^). (**c**) Correlation of *k*_*d*_ returned from fitting Eq. (4) versus the corresponding true values used in generating the parent surrogate data, where both kinetic constants were constrained as global values for each curve set. Each curve set contained three replicate injections of 1 mM **A**, performed at flow rates 0.1 m/s, 0.01 m/s, 0.001 m/s, respectively. *k*_*t*_ was determined at each flow rate using blank inhibition curves, where **A** = 0, and the relation *k*_*t*_ = *t*_c_*u^1/3^ allowed a flow rate-independent mass transport coefficient *t*_*c*_ to enable global estimation of *k*_*t*_. (**d**) Correlation of *k*_*d*_ with *k*_*a*_ for the analysis given in (**c**). (**e**) Divergence of response-normalized dissociation curves over a range in R_0_/R_max_, where **A** = 0. Four curves representing four R_0_/R_max_ values of 0.125, 0.250, 0.500, 1.000, were simulated. The curves were superimposed by normalization with respect to maximum response allowing divergence in the dissociation profile to be visualized. (**f**) Relative error in kinetic parameters returned from fitting Eq. (4) to surrogate experimental data over a wide range in R_0_/R_max_. Performed as described in (**c–d**) but over a range in R_0_/R_max_ values of 0.0156, 0.0312, 0.0625, 0.125, 0.250, 0.500, 1.000. Fitting of Eq. (4) was also performed with *k*_*d*_ held constant at its true value and with *k*_*a*_ held constant at its true value to reveal systematic error in *k*_*d*_ estimation. Plots (**c**, **d**, **f**) include error bars ± parameter fitting error but are low in magnitude and are not visibly resolved from each plotted data point at these font settings. Note that parameter-fitting error is a measure of the amount of information in the curves available to define a unique parameter value and is distinct from parameter recovery error, which compares the estimated value to the true value. It is possible to return low parameter fitting error while parameter recovery error may be relatively high.

In principle, it is possible to maintain inhibition of rebinding when *k*_*d*_ > *k*_*t*_ through rapid alkylation of the inhibition complex, where *k*_*inact*_ > *k*_*d*_ and *k*_*inact*_ > *k*_*t*_, leading to a partitioning function z = (1 + *k*_*inact*_/*k*_*t*_). However, such high *k*_*inact*_ values are highly unfavorable in drug discovery due to their non-specific alkylation potential and therefore we consider only *k*_*inact*_ < 1 s^−1^, allowing z to be neglected. Therefore, estimation of *k*_*a*_ and *k*_*d*_ for transient irreversible binding remains identical to reversible inhibitors. To illustrate this, we added an irreversible inhibition complex (AB*) to the computational model such that formation of an irreversible inhibition complex (AB*) proceeds at rate constant *k*_*inact*_, where dAB*/dt = *k*_*inact*_.**AB**. Inhibition curves at six *k*_*a*_ values were replicated (n = 8), at four transient binding levels (0.1 ≤ *k*_*d*_ ≤ 10 s^−1^) with, and without, inclusion of irreversible alkylation (*k*_*inact*_ = 1 s^−1^ ) for a total of forty eight separate conditions. As shown in Fig. 5a, the resulting forty eight inhibition curves superimpose almost perfectly at each *k*_*a*_ value, indicating that the rebinding assay may be expected to return *k*_*a*_ estimates using Eq. (4) without inclusion of a partition function while *k*_*t*_ >  > *k*_*d* ,_ or *k*_*t*_ >  > *k*_*inact*_ hold.

As shown in Fig. 5b, inhibition of rebinding will decrease for transient inhibitors when *k*_*t*_ is in the same order, or less than *k*_*d*_ and follows a partition function ${\text{ f }} = \frac{1}{{1{ } + { }k_{d} ./k_{t} }}$, where total hydrogel escape is β = *k*_*t*_ + f.*k*_*a*_.**A**_._ It also implies that the measurable *k*_*d*_ -range may be increased by employing conditions that promote a wider range in *k*_*t*_, (see Eq. (2)) and modulating secondary non-specific transient interactions between the hydrogel and the target. The surrogate experimental data in Fig. 5c,d assume a dissociation phase beginning at 10% of saturation R_0_ = 0.1.R_max_ and exhibit low systematic error (< 5%) over 2-orders for simultaneous estimation of *k*_*a*_ and *k*_*d*_. Higher R_0_/R_max_ results in higher measurement error, as shown in Fig. 5e,f. Multiple dissociation phase curves were generated over a range in R_0_/R_max_, were response-normalized (Fig. 5e) and show a maximum divergence of ~ 6% occupancy at R_0_/R_max_ = 1 with negligible divergence for R_0_/R_max_ ≤ 0.25. Propagation of this systematic deviation into error in kinetic parameter return was evaluated by fitting Eq. (4) to surrogate experimental data over a wide range in R_0_/R_max_, as shown in Fig. 5f. The lowest systematic error was observed for *k*_*a*_ -estimation when *k*_*d*_ was held constant, resulting in a maximum of 13% underestimation at R_0_/R_max_ = 1, while < 4% error was observed at R_0_/R_max_ < 0.2. This error is related to divergence of the dissociation curves, when R_0_/R_max_ > 0.2, causing a ~ twofold increase in systematic deviation when both *k*_*a*_ and *k*_*d*_ were fit simultaneously, though this remains within an acceptable error range (< 1.3-fold error) for drug discovery applications. These results are expected as the magnitude of heterogeneous rebinding regimes^20^ exhibiting multiphasic dissociation increases when dissociation traverses a wider occupancy range.

### Limit of detection and parameter return for rebinding assay relative to competitive kinetics

**Figure 6:**
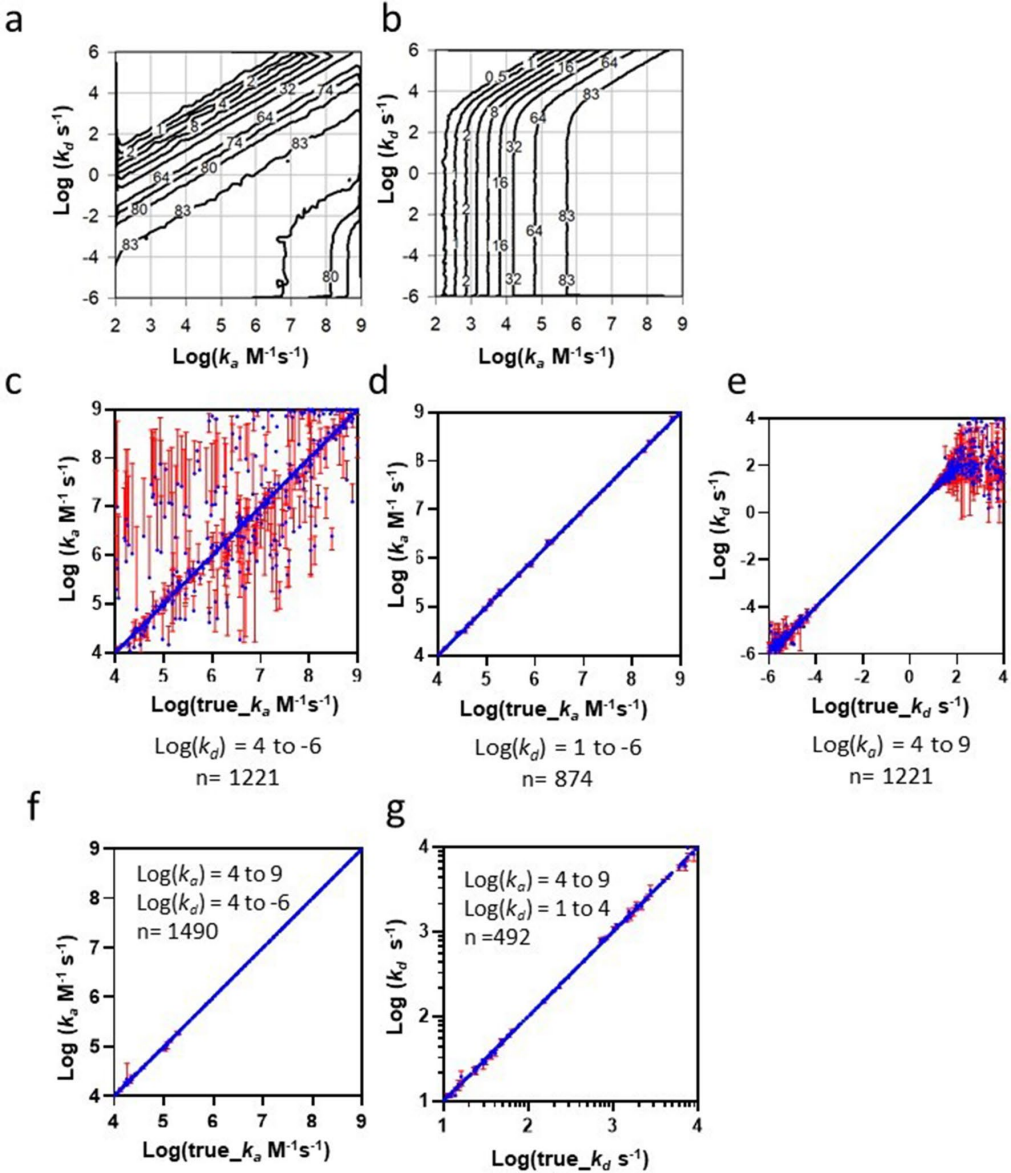
Comparison of the competitive kinetics assay with the rebinding assay in terms of sensitivity and parameter estimation, evaluated using Monte Carlo simulations seeded with pairs of pseudo-random kinetic values. (**a**) Affinity space plot for competitive kinetics with contour curves connecting regions of equal response (response (RU) is inset on contour curves). (**b**) As in (**a**) but replicated for the rebinding assay format. (**c**) Correlation of *true_k*_*a*_ versus *k*_*a*_ returned from fitting the competitive kinetic model, associated with the parent Monte Carlo simulation, for pseudo-random *k*_*a*_/*k*_*d*_ combinations spanning 10-orders with limits 4 ≤ Log(*k*_*a*_) ≤ 9 and − -6 ≤ Log(*k*_*d*_) ≤ 4. The diagonal, or unit slope, indicates the accuracy of parameter return and the SE associated with the parameter fit is indicated by the ± error bars. (**d**) Data set given in (**c**) were *k*_*a*_*/k*_*d*_ combinations containing transient *k*_*d*_ values were eliminated by restricting the *k*_*d*_ limit to − -6 ≤ Log(*k*_*d*_) ≤ 1. (**e**) As in (**d**) but given in terms of the fitted *k*_*d*_ versus the true-*k*_*d*_. (**f**) Correlation of fitted *k*_*a*_ versus the *true_k*_*a*_ for the rebinding assay format. Assay parameters for the Monte Carlo simulations were matched with competitive kinetics in (**c**) and are shown over the full kinetic range given by limits 4 ≤ Log(*k*_*a*_) ≤ 9 and − 6 ≤ Log(*k*_*d*_) ≤ 4. (**g**) Correlation of fitted *k*_*d*_ versus the *true_k*_*d*_ for the rebinding assay in (**f**), where *k*_*d*_ was restricted to transient *k*_*d*_ values over the limit 1 ≤ Log(*k*_*d*_) ≤ 4. See methods section for more details.

Monte Carlo simulations seeded with pairs of pseudo-random kinetic values were generated to compare the solution phase competitive kinetic binding model of Motulsky-Mahan^21^ with the rebinding assay given by Eq. (4). For each assay format, the iso-response contours on the top left-hand corner define the sensitivity limit and indicate a broad measuring range. For competitive kinetics shown in Fig. 6a, the diagonal iso-response contours are affinity isotherms that dominate affinity space while vertical iso-response contours are confined to an affinity region composed of tightly bound inhibitors (bottom right-hand corner), indicating *k*_*a*_ -driven inhibition. Conversely, as shown in Fig. 6b, vertical iso-response contours are observed for the rebinding assay indicating fully *k*_*d*_ –independent *k*_*a*_ –determination over the majority of affinity space. Affinity isotherms are confined to extremely transient affinity space because such transient complexes approach steady-state faster than the inhibitor can escape the hydrogel and become subject to partition function f.

The kinetic measuring range of both formats were evaluated using Monte Carlo simulations^22^ where each respective kinetic model was back-fit to a large set of simulated curve sets produced from each respective parent model and is shown in Fig. 6c–g. For competitive kinetics, the *k*_*a*_ -correlation plot in Fig. 6c shows that kinetic parameters are poorly defined over a broad range in *k*_*a*_ when *k*_*d*_ is transient, while the remainder of the simulations returned reliable *k*_*a*_ estimates, as shown in Fig. 6d. Furthermore, the *k*_*d*_ -correlation plot in Fig. 6e also shows poor *k*_*d*_ estimates for transient binders. In contrast, the *k*_*a*_ -correlation plot for the rebinding assay shown in Fig. 5f indicates that *k*_*a*_ remains well defined over the full *k*_*a*_ -range and included highly transient binders. In addition, the associated *k*_*d*_ -correlation plot shown in Fig. 6g indicates that reliable *k*_*d*_ estimates are returned for transient binders, consistent with the results obtained for surrogate data generated from the full computational model and fitted to Eq. (4), as shown in Fig. 5d,f.

## Discussion

The measurement of transient inhibitor kinetics in a practical drug discovery setting has not been enabled despite three decades of real-time, label-free technologies. For example, early chemical matter is often transiently bound, with rapid development of a steady-state plateau that is devoid of kinetic information. In general, steady-state dose response curves are prone to artifacts and it is self-evident that mechanistic discrimination of high quality hits based on exceeding a threshold *k*_*a*_ would be valuable in prioritizing compounds. The time required to fully displace the volume of the flow cell (> 0.1 s) defines the upper kinetic limit of available biosensors and system modifications that overcome this limit are not yet commercially available^11^. This longstanding unmet need has been addressed here without ultra-fast injection/detection systems by exploiting analyte rebinding within a crowded receptor environment, mixed-phase partitioning in a flow injection configuration and development of an algebraic model (Eq. (4)) of these interdependent processes.

A practical rebinding model for flow injection-based biosensors does not exist and is complicated by the many physical parameters that define the overall system. For example, thick hydrogels containing high concentrations of binding sites may be required for the rebinding assay and a hydrogel resistance term Tγ^17^ is needed (see Eq. (2)) to account for the associated increase in mass transport resistance. Equations (1–3) show that rebinding is critically dependent on *k*_*t*_ yet it remains impractical to pre-estimate because it requires precise estimates of hydrogel parameters (e.g. H_gel_, D_gel_, K_part_) that are usually unavailable. In conventional biosensing, rebinding is an interference that compromises kinetic analyses and injection of a soluble form of bound ligand during the dissociation phase has occasionally been employed to inhibit rebinding in an attempt to recover the true dissociation rate constant from simple 1:1 binding curves. Indeed a semi-analytical numerical model was reported^23^ for this rate recovery application but was unsuitable for more general applications. While Eq. (4) has been developed for kinetic analysis of inhibitor-target interactions, it may also be employed for this rate recovery application by pre-estimating *k*_*a*_, *k*_*d*_ and *k*_*t*_ while solving for *k*_*d*_*′*.

Equation (4) assumes a phenomenological encounter complex^24^ that is partitioned between alternative reaction paths and follows from a recently reported self-rebinding model^25^ for bulk solution phase interactions. Equation (4) extends beyond self-rebinding by accounting for rebinding within a receptor-crowded hydrogel and with mixed-phase partitioning in a flow injection configuration. In common with the well know two-compartment model^15^, *k*_*t*_ accounts for mass transport resistance but in our rebinding configuration target is partitioned between surface rebinding and escape as a function of *k*_*d*_*/ k*_*t*_, thereby providing extraordinary sensitivity to transient kinetics (e.g. ≤ ms-time domain). This obviates the need for ultra-fast opto-electronics and enables *k*_*d*_ -independent estimates of *k*_*a*_ for non-transient complexes. Partitioning is strongly dependent on hydrogel dimensions and relative spacing of interactants. Briefly, the escape rate of **B** and **AB** through the diffusion boundary layer is *k*_*t*_ and the probability that **B..P** reforms **BP** before exiting a hydrogel of height *H*_*gel*_ is a function of the mean free distance d traveled by liberated **B** before being rebound^17^ and is given by P_r_ = 1-exp[-(H_gel_/d)], where d = (D_gel_.*K*_*part*_/(* k*_*a*_*′*.**P**))^0.5^. In the case that d <  < H_gel_, as for a thick hydrogel and/or a high reaction flux coefficient (*k*_*a*_*′.P*), then P_r_ ≈ 1 and reformation of **BP** is favored. However, when d >  > *H*_*gel*_ then P_r_ ≈ 0 and escape of **B** from the hydrogel is favored.

In agreement with Eqs. (1) and (2), surrogate-rebinding data showed that *k*_*t*_ increased exponentially with decreasing hydrogel thickness H_gel_ and increased at higher flow velocities with expected ν_c_^1/3^ scaling. The rebinding model returns *k*_*a*_ estimates that are fully independent of steady-state, or *k*_*d*_ , while *k*_*d*_ <  < *k*_*t*_ , and this condition was adopted for analysis of the surrogate-rebinding assay data in Fig. 3. The two curve sets in Fig. 3a share identical simulation parameters other than *k*_*a*_*′*, giving a tenfold difference in mass transport limitation. The analysis shows that the rebinding assay measured inhibitor kinetics over a wide range (2.5–3.5 orders) at a single test concentration (Fig. 3a,b). The analysis supports operation in a moderate mass transport limited regime since this allowed inhibition to occur at lower inhibitor concentrations (Fig. 3b) thereby avoiding compound solubility artifacts. This elevated parameter return error (Fig. 3b,c) but *k*_*a*_ estimates were nevertheless returned with < 1.2-fold relative error and with narrow (< 5%) confidence limits (assuming 95% confidence interval). While the primary objective of the current work was to develop, and validate, Eq. (4) using surrogate data from the computational model, we also demonstrate an experimental proof-of-principle as shown in Fig. 4. Indeed, *k*_*a*_ estimated from Eq. (4) was in good agreement with estimates from direct binding and was cross-validated within the rebinding assay itself by also introducing the probe as the inhibitor species. Experimentally, this proof-of-principle shows that the rebinding assay format is comparable to direct SPR binding in terms of experimental complexity and data analysis. As already mentioned, when *k*_*d*_ <  < *k*_*t*_, then **AB** is relatively stable such that it remains independent of both *k*_*d*_ and any slower coupled kinetic reaction rates. The data in Fig. 5a implies that *k*_*a*_ may be measured for complex interaction mechanisms, such as irreversible inhibition, providing an opportunity to complete full mechanistic analysis with estimation of the fundamental kinetic rate constants (i.e. *k*_*d*_, *k*_*a*_, *k*_*inact,*_). However, when *k*_*d*_ = *k*_*t*_ then affinity partitioning results in 50% loss in inhibition (Fig. 5b) and transient partitioning ultimately defines the limit of detection of the assay. However, such partitioning is sensitive to transient kinetics allowing *k*_*a*_ and transient *k*_*d*_ to be measured over 2-orders (Fig. 5c,d). Furthermore, while the assay performs best when R_0_ < 0.1.R_max_, it was found that holding R_0_ = R_max_ (Fig. 5e,f) incurred < 1.3-fold relative error in kinetic estimates, an acceptable tolerance for many drug discovery applications.

Solution-phase competitive binding kinetics may be described by the analytic model of Motulsky-Mahan^20^ and assumes formation of a “hot” inhibition complex containing a tracer compound to indirectly report the evolution of the inhibition complex. This format can be replicated for surface sensitive biosensors^13^ by injecting a mixture containing soluble probe and inhibitor over a target-coated surface to generate a resolvable binding response, assuming a significant refractive index difference exists for binding of the competing probe relative to inhibitor binding. In solution-phase competitive kinetics, the reactions evolve towards steady-state, whereas the rebinding format maintains a *k*_*a*_-driven regime over a wide range. To explore these contrasting properties, sensitivity analysis was performed for both formats using Monte Carlo-like simulations seeded with random kinetic parameter values, and the results are shown in Fig. 6a,b. The resulting affinity plots show that the rebinding assay reports *k*_*a*_ independently of affinity since the affinity isotherms that are typical of competitive kinetics (Fig. 6a) are absent for rebinding (Fig. 6b), except in the case of transient binders, which are detected at 33-fold higher sensitivity. Furthermore, transition from an affinity-dependent regime to an affinity-independent regime can only occur for solution-phase competitive kinetics when the inhibition complex is extremely stable (*k*_*d*_ < 1 × 10^−4^ s^−1^). For the rebinding assay, the affinity-independent regime dominates and can be further extended by increasing *k*_*t*_ and can be accomplished by limiting self-self-interactions, non-specific hydrogel interactions, and enhancing convective/diffusive mass transport within the hydrogel-flow cell system. Monte Carlo simulations were also performed to test the limits of kinetic parameter return. The data showed that solution phase affinity (Fig. 6c–e) did not return reliable kinetics for transient binding (*k*_*d*_ > 10 s^−1^), whereas the rebinding format accurately returned both *k*_*a*_ and *k*_*d*_ for such transient binders (Fig. 6f, g), while reporting affinity independent *k*_*a*_ for non-transient binding. In summary, a rebinding assay exploiting a flux balance of target partitioned between rebinding and escape from within a hydrogel film was developed. The method is well suited to resolving both *k*_*a*_ and *k*_*d*_ of transient affinity complexes with adequate throughout for practical applications, which remains challenging in a contemporary drug discovery setting. An experimental proof-of-principle demonstrated estimation of *k*_*a*_ that was independent of both *k*_*d*_ and steady-state thereby establishing the feasibility of measuring extremely rapid association kinetics for non-transient binding complexes. A comprehensive application of this method in drug-discovery is currently in progress and will be the subject of a publication in the near future.

## Methods

### Experimental proof-of-principle for estimating ***k***_***a***_ of non-transient inhibitors

Assays were conducted using a Biacore S200 (GE Healthcare Bio-Sciences AB, SE-751 84, Uppsala, Sweden) with analysis temperature set to 20 °C. All binding curves were acquired and plotted at 40 Hz and the baseline noise was approximately 0.03 RU (root mean square) thereby producing a high signal-to-noise ratio for all binding curves. All reagent coupling kits and sensors were from GE Healthcare. A biotinylated-avi-tagged 22 kDa target protein was expressed recombinantly and purified in-house using standard protocols. The probe molecule (M_r_P_ = 737 Da) was a PEGylated compound with moderate affinity for the target where a terminal primary amine on the PEG linker allowed coupling to a CM5 sensor chip through standard EDC/NHS covalent linkage chemistry. All experiments were performed using an assay buffer containing 50 mM 4-(2-hydroxyethyl)-1-piperazine-ethane-sulfonic acid (HEPES), pH 7.5, containing 0.15 M sodium chloride, 0.2 mM tris(2-carboxyethyl)phosphine (TCEP), 0.1% polyethyleneglycol (M_n_ ~ 4 kDa) and 1 mg/ml carboxymethylated dextran (M_n_ ~ 10 kDa).

Kinetics of probe binding to tethered target. Target was captured onto a sensing surface of a series S SA sensor chip. Probe was injected for 30 s in duplicate over a serial doubling dilution range from 1 µM to 31 nM prepared in assay buffer. The binding curves were double referenced and fit to a two-compartment 1:1 interaction model for estimation of *k*_*a*_ and *k*_*d*_. All parameters were fit globally.

Probe coupling. Channels 2 and 4 of a series S CM5 sensor chip were activated in-situ for 8 min using standard EDC/NHS. The chip was undocked, rinsed with buffer and 40 μl of probe solution containing 1 mM probe, diluted in a 1:1 (v/v) solution of DMSO:1 M HEPES( pH 7.5), was pipetted onto the sensing surface and incubated for 2 h at room temperature. The surface was rinsed in buffer and blocked with 1 M ethanolamine for 10 min. The chip surface was rinsed with 100% DMSO, 20 mM NaOH and ultrapure water, dried, and re-docked.

Kinetics of target binding to tethered probe. Target protein was diluted in assay buffer and was injected for 60 s in duplicate over a serial-doubling dilution range from 1 µM to 31 nM. The binding curves were double referenced and fit to a two-compartment 1:1 interaction model for estimation of kinetics. All parameters were fit globally.

Rebinding assay curves. A fresh probe-coated CM5 sensor chip was prepared, using the above probe coupling method but with a 20 min probe-solution contact time, which lowered the R_max_ relative to the previous 2 h exposure. 500 nM target was injected over channel 2 (probe-coated) at 30 μL/min for 60 s followed by dissociation. This was repeated (8 replicates) and the target was allowed dissociate from the surface between replicate cycles. The inhibitor (i.e. soluble probe) was injected over channels 1 and 2, in duplicate, at 0 μM, 0.016 μM, 0.6 μM and 4 μM with assay buffer as diluent. This inhibitor injection commenced for each of these target-loading injections when the dissociation response had decreased to 118 RU.

### Simulation details associated with Fig. 2

Mass transport limited binding curves for both association and dissociation phases were stimulated using Biaevaluation 4.1.1 (Cytiva life sciences) by selecting a two-compartment mechanistic interaction model and numerical integration of the associated coupled set of ordinary differential equations (ODE) given as

$$\begin{array}{*{20}l} {{\text{B}}\left( {{\text{solution}}} \right) = {\text{Conc}}} \hfill \\ {{\mathbf{B}}\left[ 0 \right] = 0} \hfill \\ {{\text{d}}{\mathbf{B}}{\text{/dt}} = k_{t} .\left( {{\text{Conc}} - {\mathbf{B}}} \right) - \left( {k_{a}^{\prime }{\mathbf{B}}.{\mathbf{P}}{-}k_{d}^{\prime } .{\mathbf{BP}}} \right)} \hfill \\ {{\mathbf{P}}\left[ 0 \right] = {\text{R}}_{{{\text{max}}}} } \hfill \\ {{\text{d}}{\mathbf{P}}{\text{/dt}} = - \left( {k_{a}^{\prime } .{\mathbf{B}}.{\mathbf{P}}{-}k_{d}^{\prime } .{\mathbf{BP}}} \right)} \hfill \\ {{\mathbf{BP}}\left[ 0 \right] = 0} \hfill \\ {{\text{d}}{\mathbf{BP}}{\text{/dt }} = \, \left( {k_{a}^{\prime } .{\mathbf{B}}.{\mathbf{P}}{-}k_{d}^{\prime } .{\mathbf{BP}}} \right)} \hfill \\ \end{array}$$

### Simulation details associated with Fig. 6

The simulation parameters for each assay format were matched in terms of R_0_, R_max_ and kinetic range. Kinetic parameter values were chosen pseudo randomly over many orders in both *k*_*a*_ and *k*_*d*_ to producing large sets of simulated response curves. The range in simulated *k*_*a*_ values were defined by the limit 4 ≤ Log(*k*_*a*_) ≤ 9 as this range represents diffusion limited association reactions with an upper limit bounded by the maximal diffusion rate in aqueous phase and a lower limit bounded by the onset of conformationally gated binding. The *k*_*a*_ was varied over 10-orders spanning the upper and lower limits of practical importance in drug discovery, with limit -6 ≤ Log(*k*_*d*_) ≤ 4. Competitive kinetic inhibition curves were simulated over 6-serial tenfold dilutions of inhibitor from 1 mM in order to compensate for intrinsically lower measuring range. Rebinding curves were simulated over three serial tenfold dilutions of inhibitor from 1 mM, where each was repeated at two injection flow rates. Mote Carlo simulations were generated using Graphpad Prism version 9.0.0 (Graphpad software LLC, 7825 Fay Avenue, Suite 230, La Jolla, CA, 92037, USA), with baseline noise of 0.06 RU added to response curves to mimic experimental baseline noise. Each simulated curve set was then back-fit to its parent model in order to test the accuracy of parameter return, measuring range and detection sensitivity. Both models were applied using matched constraints when fitting. *k*_*a*_ and *k*_*d*_ were fit globally and *k*_*t*_ was fixed. The number of simulations included in each plot varies because the simulation range exceeded the desired ranges a limitation of the adapted Monte Carlo routine. All parameters were fixed in both simulations other than *k*_*a*_ and *k*_*d*_. In a drug discovery setting, the minimal resolvable response change defines the limit of detection for weak binding compounds and extending this range is of considerable value. Only the highest concentration of 1 mM was required for sensitivity analysis, where the response was measured over an average of 4 points at the end of each 5 min injection and a cut-off response of 0.5 RU (signal-to-noise ratio = 8) was selected. The resulting responses were plotted as response isotherms on an affinity space plot where x-axis = Log(*true-k*_*a*_) and y-axis = Log(*true*-*k*_*d*_).

### Virtual instrument

The specifications given apply to all simulation data unless otherwise stated.

Parameter settings. Parameter values were selected within conventional operational regimes and do not represent a particular commercial instrument or surface chemistry. R_0_ ≈ 0.1.R_max_ (RU) , **A** = 1 mM, *k*_*a*_ = 1 × 10^6^ (M^−1^ s^−1^), *k*_*d*_ = 0.001 (s^−1^), *k*_*a*_*′* = 1 × 10^7^ (M^−1^ s^−1^), *k*_*d*_*′* = 0.05 (s^−1^), *k*_*inact*_ = 1 (s^−1^), M_r___B_ = *30* (kDa), M_r___A_ = *200* (Da), **P** = 1 mM (equivalent to R_max_ = 3,000 RU when fully saturated by **B**).

Instrument settings. ν_c_ = 0.1 m/s, flow cell height (h) = 20 µM, flow cell length (l) = 0.5 mm, sensing region = hydrogel domain = 0.2 mm × 200 nm. Detection reports the average concentration of a given species within the hydrogel domain. The three-dimensional hydrogel is well approximated using a two-dimensional geometry because contributions near the flow cell walls can be neglected. This is the case because the flow cell is thin relative to its width. The data collection rate was 1 Hz and baseline noise equivalent to 0.03 RU (root mean square) was added to response curves to mimic actual instrument performance.

Hydrogel modeling. Hydrogels are highly complex environments making it difficult to accurately determine species diffusion rates and related mass transport effects. For example, diffusion might be > tenfold slower at high protein concentrations and some size exclusion may also occur further slowing diffusion. For example, a mass equivalent to > 10,000 RU of protein can readily be bound within a hydrogel. This would correspond to a volume fraction of > 10% (v/v), assuming a specific volume *ϕ*_V_ (ml/g) = 0.754 and would be expected to increase viscosity > tenfold and decrease diffusion by at least this factor^26^. A 200 nm thick hydrogel was modeled as an area containing a homogenous density of hydrogel grafted to the sensing surface that decreases rapidly at the hydrogel-liquid interface according to a hydrogel density function density = 1-Exp(-100*(1-z)), where z is the dimensionless relative height of the hydrogel. The concentration of **P** is assumed to be scaled by the hydrogel density and the diffusion coefficient of all species tethered to the hydrogel (i.e. **P**, **BP**) is assumed to be zero. Diffusion of all unbound species inside hydrogel is assumed to be twofold lower due to a twofold increase in viscosity within the hydrogel relative to the bulk liquid and soluble species are subject to molecular weight-dependent partitioning. Therefore, parameters related to mass transport of soluble species inside the hydrogel are defined as follows. Diffusion coefficient of **A** = D_A_ = 5 × 10^−10^ (m^2^/s), diffusion coefficient of **B** = D_B_ = D_A_(M_r___B_ / M_r___A_)^1/3^*,* diffusion coefficient of **A** inside hydrogel = D_gel_ = 2.D_A_.K_part_, where the hydrogel partition coefficient for **A** = K_part_ = Exp(-10^−3^* M_r_A_^2/3^)*,* diffusion coefficient of all soluble species containing **B** (i.e. **B**, **AB**, AB*) inside hydrogel = D_gel_ = 2.D_B_.K_part_, where the hydrogel partition coefficient for **B** = K_part_ = Exp(-10^−3^* M_r_B_^2/3^)*.* The initial conditions for the simulation include addition of tethered **P** some fraction of which is in the form of affinity complex **BP** before the onset of the inhibitor injection. The inhibitor injection was simulated as a sample pulse entering from one end of the rectangular flow cell and exiting at the opposite end, where the tethered hydrogel film is located at one of the flow cell walls and is parallel to the direction of flow.

Finite element analysis. Coupled ODEs were solved numerically coupled to the master equations, which are partial differential equations (PDEs) governing flow, advection, diffusion, and reaction for a 20 µM thick flow cell housing a sensing region containing a hydrogel film functionalized with **P**. The entire geometry was discretized in space and solved over incremental time periods to generate surrogate experimental data. Comsol multiphysics 5.1 (COMSOL AB, Tegnérgatan 23, SE-111 40, Stockholm, Sweden) was used to perform all numerical simulations. A computational model replicating the flow injection-based biosensor system depicted in Fig. 1 was created. Typical microfluidic channels employed in biosensors have high aspect ratios where side walls are far apart relative to the top/bottom walls allowing microchannel width to be neglected reducing the model to a cross-section through the microchannel. The two dimensional flow cell geometry housing a hydrogel film grafted to the flow cell sensing region was meshed with > 14 k elements. This mesh was optimized until no detectable change was observed in the simulation output and included a higher density of elements at the hydrogel interfacial boundaries arriving at 6762 elements over the hydrogel domain. The incompressible form of the Navier–Stokes equation was used to solve the two-dimensional velocity profile through the channel, assuming steady-state, at constant flow rate and at atmospheric pressure. The initial velocity at the walls u_wall_ = 0, the inlet velocity was variable and was defined by solving for the velocity vector field over the full domain

5 $$\uprho {\text{u}} \cdot \nabla {\text{u }} = - \nabla {\text{p}} +\upmu \nabla^{2} {\text{u}}$$

where ρ is the density, p is the pressure and μ is the dynamic viscosity.

The flow velocity vector field was coupled to the steady-state advection/diffusion equation for each dilute species to solve for the analyte concentration field in the bulk flow domain and hydrogel domain.

6 $$\nabla \cdot \left( { - {\text{D}}\nabla {\text{c}}} \right) + {\text{u}} \cdot \nabla {\text{c}} = {\text{R}}$$

Here D is the diffusion coefficient, c is the concentration of a given species and R is a reaction term associated with that species. Initially the analyte concentration in the microchannel c = 0. At the inlet the initial analyte concentration profile along the microchannel height was defined by multiplying the concentration by a rectangular function to simulate a continuous injection of sample for a given contact time. **P** was assumed to be distributed within the hydrogel domain tethered to the sensing surface. The associated reactions within the hydrogel domain between soluble species with **P** distributed within the hydrogel and the formation of non-tethered affinity complexes **AB** were defined as ODEs coupled to the advection/diffusion Eq. (6) and are given as

$$\begin{array}{*{20}l} {{\text{d}}{\mathbf{A}}{\text{/dt}} = - k_{a} .{\mathbf{A}}.{\mathbf{B}} + k_{d} .{\mathbf{AB}}} \hfill \\ {{\text{d}}{\mathbf{B}}{\text{/dt}} = - k_{a} .{\mathbf{A}}.{\mathbf{B}} + k_{d} .{\mathbf{AB}} - k_{{a^{\prime } }} .{\mathbf{P}}{\mathbf{.B}} + k_{{d^{\prime } }} .{\mathbf{BP}}} \hfill \\ {{\text{d}}{\mathbf{AB}}{\text{/dt}} = k_{a} .{\mathbf{A}}.{\mathbf{B}}{-}k_{{\text{d}}} .{\mathbf{AB}}} \hfill \\ {{\text{d}}{\mathbf{P}}{\text{/dt }} = \, - k_{{a^{\prime } }} .{\mathbf{P}}.{\mathbf{B}} + k_{{d^{\prime } }} .{\mathbf{BP}}} \hfill \\ {{\text{d}}{\mathbf{BP}}{\text{/dt }} = k_{{a^{\prime } }} .{\mathbf{P}}.{\mathbf{B}} - k_{{d^{\prime } }} .{\mathbf{BP}}} \hfill \\ \end{array}$$

where *k*_*a*_ and *k*_*d*_, are the forward and reverse kinetic interaction constants for the **AB** complex and *k*_*a*_*′* and *k*_*d*_*′*, are the forward and reverse kinetic interaction constants for the **BP** affinity complex. This set of ODEs describe reversible affinity complex formation and formation of an irreversible complex (AB*) required the following ODEs

$$\begin{array}{*{20}l} {{\text{dA/dt}} = - k_{a} .{\text{A}}.{\text{B}} + k_{d} .{\text{AB}}} \hfill \\ {{\text{d}}{\mathbf{B}}{\text{/dt}} = - k_{a} .{\mathbf{A}}.{\mathbf{B}} + k_{d} .{\mathbf{AB}} - k_{{a^{\prime } }} .{\mathbf{P}}.{\mathbf{B}} + k_{{d^{\prime } }} .{\mathbf{BP}}} \hfill \\ {{\text{d}}{\mathbf{AB}}{\text{/dt }} = k_{a} .{\mathbf{A}}.{\mathbf{B}}{-}k_{d} .{\mathbf{AB}} - k_{inact} .{\mathbf{AB}}.} \hfill \\ {{\text{d}}{\mathbf{P}}{\text{/dt }} = \, - {\text{k}}_{{{\text{a}}^{\prime } }} .{\mathbf{P}}.{\mathbf{B}} + k_{{d^{\prime } }} .{\mathbf{BP}}} \hfill \\ {{\text{d}}{\mathbf{BP}}{\text{/dt }} = k_{{a^{\prime } }} .{\mathbf{P}}.{\mathbf{B}} - k_{{d^{\prime } }} .{\mathbf{BP}}} \hfill \\ {{\text{d}}{\mathbf{AB}}*{\text{/dt }} = k_{inact} .{\mathbf{AB}}} \hfill \\ \end{array}$$

The time-dependent change in species accumulation was found from a species flux balance over the hydrogel domain and the simulation was performed in time-stepping mode in order to produce reaction progress curves. The accumulation of affinity complex was expressed in terms of an equivalent biosensor response where 100 RU = 1 mg/ml of a given species.

### Curve fitting statistics

Microsoft Excel and Biaevaluation (GE Healthcare Bio-Sciences AB) were employed for data processing. Graphpad Prism version 9.0.0 (Graphpad software LLC, 7825 Fay Avenue, Suite 230, La Jolla, CA, 92,037, USA) was employed for all plots other than Fig. 6 a,b, which were ploted using DPlot Version 2.3.5.7 (HydeSoft Computing, LLC). Graphpad Prism enabled fitting of binding interaction data to interaction models by nonlinear regression, and the associated statistical methods to confirm goodness of fit and confidence in parameter estimates are well established^27^. Statistical parameters such as the standard error of the fit (SE) associated with a given parameter returned in the fit were used to report confidence in parameter estimates. The SE is a measure of the information content of the data and specifies the degree to which the curves define the parameter value from the fit. Values < 5% indicate high confidence and values > 10% indicate that the parameter is poorly defined. The goodness of fit between a model curve and an experimental curve is described by χ^2^ when the number of data points is high and by a regression coefficient R^2^ when the number of values is low. %χ^2^ is the square of the averaged residual response difference expressed as a percentage of maximum response recorded for the curve set. Typically high quality fits will produce χ^2^ values < 5%. Occasionally χ^2^ may be within acceptable limits but the fit may remain questionable if residuals are not distributed randomly. Curves generated by numerical simulation follow deterministic algorithms and therefore do not require replicates. Global parameter fitting refers to constraining a parameter value to a single global value over the entire curve set.

## References

[1] McGovern SL, Caselli E, Grigorieff N, Shoichet BK (2002). A common mechanism underlying promiscuous inhibitors from virtual and high-throughput screening. J. Med. Chem..

[2] Sink R, Gobec S, Pecar S, Zega A (2010). False positives in the early stages of drug discovery. Curr. Med. Chem..

[3] Torosyan H, Shoichet BK (2019). Protein stability effects in aggregate-based enzyme inhibition. J. Med. Chem..

[4] Copeland R (2016). The drug-target residence time model: A 10-year retrospective. Nat. Rev. Drug Discov..

[5] Sykes DA (2017). Extrapyramidal side effects of antipsychotics are linked to their association kinetics at dopamine D2 receptors. Nat. Commun..

[6] Amaral M (2017). Protein conformational flexibility modulates kinetics and thermodynamics of drug binding. Nat. Commun..

[7] Vauquelin G (2016). Effects of target binding kinetics on in vivo drug efficacy: Koff, kon and rebinding. Br. J. Pharmacol..

[8] Rich RL (2009). A global benchmark study using affinity-based biosensors. Anal. Biochem..

[9] Papalia GA (2006). Comparative analysis of 10 small molecules binding to carbonic anhydrase II by different investigators using Biacore technology. Anal. Biochem..

[10] Burton RL, Hanes JW, Grant GA (2008). A stopped flow transient kinetic analysis of substrate binding and catalysis in *Escherichia coli* D-3-phosphoglycerate dehydrogenase. J. Biol. Chem..

[11] Quinn JG, Steffek M, Bruning JM, Frommlet A, Mulvihill M (2019). Unlocking latent kinetic information from label-free binding. Sci. Rep..

[12] Georgi V, Dubrovskiy A, Steigele S, Fernández-Montalván AE (2019). Considerations for improved performance of competition association assays analysed with the Motulsky–Mahan's “kinetics of competitive binding” model. Br. J. Pharmacol..

[13] Karlsson R (1994). Real-time competitive kinetic analysis of interactions between low-molecular-weight ligands in solution and surface-immobilized receptors. Anal. Biochem..

[14] Jankovics H, Kovacs B, Saftics A (2020). Grating-coupled interferometry reveals binding kinetics and affinities of Ni ions to genetically engineered protein layers. Sci. Rep..

[15] Myszka DG, He X, Dembo M, Morton TA, Goldstein B (1998). Extending the range of rate constants available for BIACORE: Interpreting mass transport influenced binding data. Biophys. J..

[16] Hansen R, Bruus H, Callisen TH, Hassager O (2012). Transient convection, and adsorption in surface-based biosensors. Langmuir.

[17] Goldstein B, Dembo M (1995). Approximating the effects of diffusion on reversible reactions at the cell surface: Ligand-receptor kinetics. Biophys J..

[18] Wofsy C, Goldstein B (2002). Effective rate models for receptors distributed in a layer above a surface: Application to cells and Biacore. Biophys. J..

[19] Pol E (2016). Evaluation of calibration free concentration analysis provided by Biacore systems. Anal. Biochem..

[20] Goldstein B, Coombs D, He X, Pineda AR, Wofsy C (1999). The influence of transport on the kinetics of binding to surface receptors: Application to cells and BIAcore. J. Mol. Recognit..

[21] Motulsky HJ, Mahan L (1984). The kinetics of competitive radioligand binding predicted by the law of mass action. Mol. Pharmacol..

[22] Metropolis N, Ulam S (1949). The Monte Carlo method. J. Am. Stat. Assoc.

[23] He X, Coombs D, Myszka DG (2006). A theoretical and experimental study of competition between solution and surface receptors for ligand in a Biacore flow cell. Bull. Math. Biol..

[24] Shoup D, Szabo A (1982). Role of diffusion in ligand binding to macromolecules and cell-bound receptors. Biophys. J..

[25] Paramanathan T, Reeves D, Friedman L (2014). A general mechanism for competitor-induced dissociation of molecular complexes. Nat. Commun..

[26] Zhang Z, Liu Y (2017). Recent progresses of understanding the viscosity of concentrated protein solutions. Curr. Open. Chem. Eng..

[27] Motulsky, H. J. & Christopoulos, A. Fitting models to biological data using linear and non-Linear regression. A practical guide to curve fitting. (Oxford University Press 2004). ISBN-10: 0195171802.

